# Training Early Literacy Related Skills: To Which Degree Does a Musical Training Contribute to Phonological Awareness Development?

**DOI:** 10.3389/fpsyg.2016.01803

**Published:** 2016-11-16

**Authors:** Sebastian Kempert, Regina Götz, Kristine Blatter, Catharina Tibken, Cordula Artelt, Wolfgang Schneider, Petra Stanat

**Affiliations:** ^1^Institute for Educational Studies, Humboldt University of BerlinBerlin, Germany; ^2^Department of Psychology, University of WürzburgWürzburg, Germany; ^3^German Youth InstituteMunich, Germany; ^4^Department of Educational Research, University of BambergGermany; ^5^Institute for Educational Quality Improvement, Humboldt University of BerlinBerlin, Germany

**Keywords:** phonological awareness, musical training, phonological training, preschool children, early literacy

## Abstract

Well-developed phonological awareness skills are a core prerequisite for early literacy development. Although effective phonological awareness training programs exist, children at risk often do not reach similar levels of phonological awareness after the intervention as children with normally developed skills. Based on theoretical considerations and first promising results the present study explores effects of an early musical training in combination with a conventional phonological training in children with weak phonological awareness skills. Using a quasi-experimental pretest-posttest control group design and measurements across a period of 2 years, we tested the effects of two interventions: a consecutive combination of a musical and a phonological training and a phonological training alone. The design made it possible to disentangle effects of the musical training alone as well the effects of its combination with the phonological training. The outcome measures of these groups were compared with the control group with multivariate analyses, controlling for a number of background variables. The sample included *N* = 424 German-speaking children aged 4–5 years at the beginning of the study. We found a positive relationship between musical abilities and phonological awareness. Yet, whereas the well-established phonological training produced the expected effects, adding a musical training did not contribute significantly to phonological awareness development. Training effects were partly dependent on the initial level of phonological awareness. Possible reasons for the lack of training effects in the musical part of the combination condition as well as practical implications for early literacy education are discussed.

## Introduction

Well-developed language competencies and literacy skills are basic requirements for academic success and social integration. Thus, for children at risk an early training of these competencies is key for promoting individual academic development. Helping children to catch up with their peers in language-related competencies as early as possible prevents disparities in subsequent phases of academic development. Not least because of the growing linguistic, cultural, and socioeconomic diversity and the associated academic heterogeneity, many countries throughout Europe as well as the U.S. make efforts to increase educational quality already in kindergarten and preschool in order to successfully prepare children for entry into primary school (e.g., European Commission, 2014). Among the measures designed to promote language-related skills, the support of early literacy development by training phonological awareness is a prominent example. Phonological awareness, as a part of metalinguistic awareness, includes the ability to detect, analyze, and manipulate sounds in oral language (Tunmer and Hoover, 1992; Lonigan, 2006). Examples are the ability to rhyme words, to segment words into syllables as well as to blend and delete phonemes. The ability to analyze and manipulate larger sound units, such as words and syllables, is labeled as *phonological awareness in the broader sense* (broad) whereas the ability to analyze and manipulate small sound units (phonemes) is referred to as *phonological awareness in the narrower sense* (narrow; Skowronek and Marx, 1989).

### Effects of phonological training programs

Phonological awareness is the most important specific predictor of literacy acquisition (Bishop and League, 2006). The skills associated with phonological awareness are of prime importance in the first steps of learning to read and write. Ultimately, verbal speech and sounds have to be broken down into discrete units (i.e., words, syllables, and phonemes) in order to assign them to letters, and reversely, written text has to be decoded by matching letters to their phonetic correspondent, as a basis for identifying syllables and words. Longitudinal studies show that children with highly developed phonological awareness tend to profit more from early literacy instruction and reach higher levels of reading competency than children with less developed phonological awareness (e.g., Whitehurst and Lonigan, 1998).

Meta-analyses on the effectiveness of phonological awareness training programs also support the view that early (preschool) literacy skills are important predictors of reading and writing development. Not only do these trainings (on average) enhance phonological awareness in children, they also improve subsequent literacy development (see Bus and van Ijzendoorn, 1999; Ehri et al., 2001). Both meta-analyses revealed strong direct effects on phonological awareness and strong to medium-sized effects on reading competencies in intervention studies that were carried out in English-speaking countries. However, the results of two recent meta-analyses in the German context indicate that the effect sizes are not necessarily consistent across different languages (Fischer and Pfost, 2015; Wolf et al., 2016). Although the German meta-analyses found direct effects of the trainings on phonological awareness as well as the transfer-effects on reading and writing skills, they were substantially lower than the effects reported by English meta-analyses. These discrepancies in the findings may be due to differences in the orthographic consistency between languages, with German having a more consistent orthography than English. More specifically, the importance of phonological awareness as a prerequisite for literacy development may depend on the extent to which the correspondence between phonemes and graphemes is consistent. Lower consistency (as in English compared to German) may require phonological awareness skills to a higher degree (Fischer and Pfost, 2015; Wolf et al., 2016).

The preschool program *Hören, Lauschen, Lernen 1*, and *2* (Hear, Listen, Learn [HLL]; Plume and Schneider, 2004; Küspert and Schneider, 2008) is a well-established German intervention program which focuses on phonological awareness in combination with a phoneme-letter training. This program has been shown to be effective in a series of empirical studies with German-speaking children (e.g., Schneider et al., 1997, 2000; Marx et al., 2005). In addition, several studies revealed that minority language children benefit from the training to a similar degree as children who only speak the majority language in their family (Weber et al., 2007; Blatter et al., 2013). However, despite showing similar gains in phonological awareness overall, the minority language children continued to lag behind their majority-language peers in the level of phonological awareness they reached after the intervention. The training could thus not fully compensate the initial disparities (see also Schöppe et al., 2013 for similar results).

In searching for measures that may help to close this gap, some limitations of the available approaches become obvious. Although the training instruments at hand appear to be effective overall, it would be difficult to raise the probability of compensatory effects in children with weak initial phonological skills by simply extending their application. There seems to be a sensitive phase of phonological awareness development, with trainings typically having the largest impact in children between 5 and 6 years of age (Bus and van Ijzendoorn, 1999; Ehri et al., 2001). To benefit from a verbal phonological training, a certain level of language proficiency, and basic linguistic knowledge are necessary for adopting a reflective perspective on linguistic phenomena (Marx et al., 2005). These prerequisites are associated with children's general development and therefore with age (Gathercole and Baddeley, 1993).

Thus, a central goal of the present research was to explore an additional, non-verbal approach to promoting phonological awareness that can be combined with the well-established verbal trainings.

### Training of phonological awareness skills with musical contents

Theoretical considerations as well as evidence from a few empirical intervention studies suggest that musical training in early childhood may enhance phonological awareness skills (e.g., Gromko, 2005; Degé and Schwarzer, 2011; Moritz et al., 2013; see also the meta-analysis by Gordon et al., 2015). McMullen and Saffran (2004), for example, suggest that music and language share the same basic processing mechanisms, especially at an early stage of child development. In both language and music only combinations of single auditory stimuli form meaningful units (e.g., a word or a motif) and the processing of the respective stimuli requires similar cognitive resources in terms of attention and memory (cf. Kraus and Chandrasekaran, 2010). In a first step, to assign single stimuli like phonemes or pitches to similar or different categories, they have to be perceived and differentiated (Patel, 2003, 2008). With increasing age, children are becoming aware of and are able to distinguish even subtle nuances within a category (e.g., same rhythms from different sources or similar combinations of phonemes from different speakers) as well as differences between categories (e.g., differences between two different rhythms or two different phonemes). The ability of perceiving, differentiating, and categorizing linguistic as well as musical stimuli in early childhood is theorized to be based on the same cognitive processes responsible for converting auditory stimuli in general. According to the *shared sound category learning mechanism hypothesis* postulated by Patel (2008), the results of learning music and language are different, but the development in both domains is based on shared cognitive processes, therefore influencing each other. Similarly, Koelsch and Siebel (2005, p. 582) argue that a common neural basis exists, as the human brain “does not treat language and music as strictly separate domains, but rather treats language as a special case of music.” Studies using functional neuroimaging techniques support this view by showing that dynamic sound processing is located in the same brain regions regardless whether the stimuli are of speech or nonspeech origin (see Tallal and Gaab, 2006, for a review). Forgeard et al. (2008) discuss further factors of the interdependence of musical and language development. For example, they assume that putting words to music during the activity of singing calls for the exaggerated accentuation of words helping children to learn to segment words into syllables. Moreover, the notation of music shares similar features involved in reading and writing activities such as mapping sound symbols or pattern recognition. Finally, the experience in listening to and producing different sounds during musical activities can be seen as an “engaging, pleasurable auditory training […]” that also improves the processing of speech sounds (Forgeard et al., 2008, p. 384). Based on these assumptions, to be engaged in either musical or phonological activities should have positive effects on phonological awareness (for a further discussion and a review of empirical results see also Asaridou and McQueen, 2013).

There are some correlative analyses corroborating the assumption that musical activities and phonological competencies are indeed positively related. Anvari et al. (2002) tested a sample of *N* = 100 children at the age of 4–5 years. They found medium to strong correlations between phonemic awareness and musical ability (measured by rhythm discrimination, chord discrimination, melody discrimination, rhythm production, and chord analysis). Even the correlation between reading skills and musical ability proved to be significant. Furthermore, findings from a hierarchical regression analysis revealed that musical awareness had a predictive effect on reading skills in its own right, even after its shared variance with phonological awareness was taken into account. Accordingly, the authors reasoned that “music perception appears to be tapping auditory mechanisms related to reading skill that only partially overlap with those related to phonological awareness” (Anvari et al., 2002, p. 122).

Lamb and Gregory (1993) examined phonological awareness, reading readiness, and musical ability in pre-readers at the age of 4–5 years. Musical ability was measured with two specifically designed subtests tapping timbre and pitch discrimination. While timbre discrimination was unrelated to all other measures, pitch discrimination correlated significantly with phonological awareness and both reading tests. Furthermore, the expected correlation between phonological awareness and early reading skill was significant.

In a recent study, Degé et al. (2015) examined the associations between productive and receptive musical abilities and precursors of reading including phonological awareness in 6-year-old preschool children. The results revealed not only significant correlations between productive and perceptive musical abilities with phonological awareness but also associations with further important precursors such as working memory and rapid retrieval of information from long-term memory.

Studies with older children provide additional correlational evidence for the interdependence of musical and phonological skills. Loui et al. (2011) present data of children aged seven to nine indicating a strong relationship between pitch perception/production skills and phonological awareness measured by the ability to categorize phonemes as well as to analyze compound words. Using a correlational design, Zuk et al. (2013) demonstrate that the relationship between musical skills and phonological awareness may indeed expand to literacy skills. Elementary school students aged 6–8 years showed significant shared variance on the applied musical sequence transcription task and literacy skills including reading speed and accuracy as well as word writing.

More conclusive evidence in terms of causality stems from training studies designed to promote phonological awareness with musical activities. Using an experimental pretest-posttest control group design, Degé and Schwarzer (2011) found that 5- to 6-year-old preschoolers benefited to a similar degree from a short-time musical training as children participating in a phonological training program (HLL). Both groups (*N* = 13–15 per group) reached higher scores in the post-test of phonological awareness than a treated control group that received training in sports. However, the effect was only observable in the awareness of larger phonological units (broad).

In another experimental study with groups of 5-year-old preschoolers (*N* = 15 per group), Moritz et al. (2013) demonstrated that the improvement in phonological awareness was related to the intensity of a musical training. Children who received intensive daily music lessons significantly improved in all six areas of phonological awareness that were investigated (discrimination and production of rhymes, segmentation of sentences and syllables, isolation of initial phonemes, and deletion of sounds). The control group, which received musical training only once a week, improved significantly less in four of the six areas and not at all in rhyming skills.

The musical training in the study by Degé and Schwarzer (2011) included a relatively broad spectrum of musical topics, such as joint singing and drumming, meter execution, training of rudimentary notation skills, rhythmic exercises, dancing, and playful familiarization with intervals. In comparison, Moritz et al. (2013) focused on rhythm (e.g., motor rhythm training with significant emphasis on rhythmical patterns, an initial emphasis on beat development, use of rhythmic entities to create new rhythmic combinations, or singing-game songs from which basic rhythm and melodic units were abstracted). Both studies used a sound experimental design, designed the training content very carefully, and took relevant background variables such as the socioeconomic status or cognitive abilities into account. However, due to the small sample sizes, the stability, and generalizability of the results are unclear.

Another intervention study, using a larger sample of *N* = 103 children at the age of 4–5 years, was conducted by Gromko (2005). The goal of this study was to test the so-called *near-transfer hypothesis* which essentially postulates “that music instruction that emphasizes the development of aural perception would lead to significant gains in the development of young children's phonemic awareness […]” (Gromko, 2005, p. 206), particularly their phoneme segmentation ability. The children in the experimental group received 4 months of music instruction for 30 min per week, whereas the control group did not receive any music instruction. Besides singing and rhythmical education with body percussion, kinesthetic movement, and playing percussion instruments, the treatment lessons in this study also included graphic charts with symbols like dots, squares, or lines representing steady beat, word rhythms, or melodic contour. Central results of the study were significant advantages of the experimental group compared to the control group especially for phoneme segmentation fluency. The major limitation of this study is its quasi-experimental design. Furthermore, important background variables, such as the socioeconomic status of children's families were not included in the statistical analyses, even though the authors reported differences between the two groups in this regard on a descriptive level.

A recent meta-analysis on the effects of musical trainings on literacy related skills demonstrated only small effects of musical trainings on phonological awareness and reading (Gordon et al., 2015). However, the authors point to the remarkable differences in the quality of studies (e.g., inclusion of background variables, sample size etc.) as well as in the outcomes indicating the need of further research on this topic.

In conclusion, there are theoretical arguments as well as empirical evidence suggesting that not only phonological trainings but also musical trainings are suited for promoting phonological awareness at an early stage of child development and hence for helping to preparing children for the successful acquisition of written language (for studies on older children and/or children with dyslexia see e.g., Flaugnacco et al., 2015; Habib et al., 2016). If this were the case, a combination of both trainings should have stronger effects on phonological awareness than a phonological training alone.

Combining the two types of training would take advantage of their respective advantages. First, it is possible to conduct musical trainings earlier than phonological trainings. There is no evidence that conventional phonological trainings are effective before 5–6 years of age and attempts to advance phonological trainings to the penultimate year of preschool (Rothe et al., 2004) point to overall smaller effect sizes for 5-year-old children compared to the 6-years-olds. However, a musical training can be designed in a way that requires less language proficiency and linguistic knowledge than a verbal phonological training. This offers the opportunity for an earlier start of intervention. Second, combining different training approaches with different contents may increase the overall attractiveness of the training as diverse interests and preferences are covered. This may have a positive impact on the engagement and motivation of trainers as well as participants (c.f. Forgeard et al., 2008; Moritz et al., 2013).

### Research questions and hypotheses

Drawing on the theoretical assumptions and the available empirical evidence described above, the present study investigated three research questions and hypotheses:

1. Does a musical training applied in the penultimate year of preschool have an impact on phonological awareness? We expect a small positive effect on phonological awareness, especially on phonological awareness (broad).
  Only a small effect is predicted for two reasons: First, theoretical considerations as well as previous studies point to the fact that training phonological awareness is quite challenging before the age of 5 years (c.f. Bus and van Ijzendoorn, 1999; Ehri et al., 2001). On average, the children participating in our study were even half a year younger (i.e., 54 months old) which means a substantial developmental difference at this age. Second, although there is evidence that musical activities may be suited for training phonological awareness, the format of phonological awareness tests differs from that used for tests tapping musical contents. Thus, some kind of transfer is required which may influence the outcome (c.f. Bransford and Schwarz, 1999 on the difficulties in research on skills transfer). Moreover, in the study by Degé and Schwarzer (2011) the musical training program was effective only for phonological awareness (broad). Apparently, the transfer effect of the musical training is most likely to be found for this aspect of phonological awareness, although there are no theoretical reasons why effects on both aspects (i.e., phonological awareness in the broad and narrow sense) should not be observed.
2. Does a consecutive combination of musical training and a well-established training of phonological awareness in the last 2 years of preschool have a stronger impact on phonological awareness (broad and narrow) than a training of phonological awareness alone? Based on the evidence of previous studies, a small to medium-sized effect of the phonological training is expected. Furthermore, a small incremental effect of the combined training is expected, resulting from the addition of the individual effectiveness of each training program.
3. Does the effect of the combined training depend on the children's initial phonological awareness skills? We expect an aptitude-treatment interaction in the sense that children with weak initial phonological awareness skills will benefit more from the two consecutive trainings than children with normally developed phonological awareness skills because of a higher developmental potential in the former group. The key question is whether a compensatory effect can be observed in children with weak initial phonological awareness skills, which is defined as reaching post-test achievement levels comparable to children with normally developed phonological awareness skills in the control group. To be precise, only an effective remediation that helps children with weak initial skills to catch up with children with normal initial skills (without training) is considered to be compensatory.

## Materials and methods

### Study design

The present study employed a quasi-experimental pretest-posttest design with four measurement points over a time period of 2 years (August 2012 to July 2014). Within these 2 years, the participating children were tested at the beginning as well as the end of their penultimate and ultimate year of preschool. At each of the four measurement points (t_1_–t_4_), we tested a range of cognitive abilities as well as language and musical competencies.

The sample was subdivided into two treatment groups and one control group. The first group took part in a training of musical skills between t_1_ and t_2_ as well as in a training of phonological skills between t_3_ and t_4_ (TG_music/phon_). The second group participated only in the training of phonological skills between t_3_ and t_4_ (TG_phon_). A third group served as the control group (CG) which only attended the regular preschool program and did not receive any special training. Between t_1_ and t_2_ the TG_phon_ was also considered to be a control group as these children did not receive any treatment before t_3_.

Some of the participating preschools chose the assignment to one of the treatment groups themselves or were assigned to one of the condition for organizational reasons. Hence, the study did not realize a randomized design. This strategy was necessary to ensure the compliance of the preschool staff with regard to the extensive training and testing procedures. Although in most facilities, more than one group participated in the study, each preschool was assigned to only one condition to avoid treatment diffusion.

### Participants

Overall, 34 preschools^1^ were recruited for the study in small, medium, and large cities in Germany. Not included were preschools that reported to conduct other phonological or musical training programs besides the ones evaluated in the present study. Further, children with learning disabilities or speech impairments (including hearing impairment) as indicated by the preschool teachers did not take part in the study. Thus, the sample of the study is not representative. Within the 34 preschools, *N* = 436 children at the beginning of their penultimate year of preschool participated in the study.

Some cases were excluded from the following analyses because of problems occurring during the training phases (e.g., early cessation of the training due to teacher illness or organizational constraints within the preschools). The following analyses are based on a sample of *N* = 424 children (51% girls) with a mean age of 54.8 months (SD 4.5 months) at the first measurement point. About 48% of the children had a minority-language background meaning that at least one of their parents speaks another first language than German.

As is typically the case in longitudinal studies, missing values occurred at all four measurement points (8% overall). To avoid loss of statistical power and biased estimates, missing data were substituted using a single imputation procedure in SPSS 22.0. We used a background model with language skills, cognitive abilities, and socioeconomic status as predictors to generate a complete dataset accounting for the training conditions and the longitudinal structure of the data. The following analyses are based on this dataset which did not differ in all central variables from the original dataset (all *p*s < 0.05). See Table 1 for characteristics of the sample of children overall and of the children assigned to the three conditions.

**Table 1:** **Characteristics (ratios, means, and standard deviations) of the overall sample as well as the three subsamples at t_**1**_**.

	**Total (*N* = 424)**	**CG (*N* = 187)**	**TG_music/phon_ (*N* = 128)**	**TG_phon_ (*N* = 109)**
Age in months	54.8 (4.5)	55.1 (4.8)	54.4 (4.0)	54.9 (4.7)
Sex (girls)	51%	49%	56%	47%
Language-minority children (yes)	48%	46%	47%	52%
Repeating sentences		71.62 (22.42)	70.70 (25.66)	66.60 (22.77)
Plural/singular composition		35.27 (20.44)	39.02 (20.35)	39.73 (19.94)
Productive lexicon		63.64 (19.11)	61.93 (19.43)	63.22 (18.11)
Receptive lexicon		62.51 (15.84)	62.25 (16.34)	61.22 (15.60)
Musical competencies		59.38 (19.09)	58.60 (19.50)	60.67 (20.57)
Nonverbal cognitive abilities		25.20 (5.67)	25.74 (5.49)	25.56 (6.53)
Working memory		1.56 (0.95)	1.55 (0.80)	1.52 (0.92)
Socioeconomic status HISEI		51.74 (17.60)	50.18 (18.11)	54.51 (19.09)

*Between t_1_ and t_2_ the CG and the TG_phon_ formed a combined control group; raw scores are displayed for nonverbal cognitive abilities, working memory, and socioeconomic status. All other scores are displayed in percentages of correct answers; HISEI = Highest International Socio-Economic Index of Occupational Status—see below for further explanation*.

To identify children with weak phonological skills at t_1_ for detailed analyses exploring the effectiveness of the training programs for this group, we chose the 25th percentile of a combined measure of phonological awareness (broad and narrow; see measures section for details on the instruments) as the selection criterion. As there is no norm-based information on when a child is considered to have weak phonological awareness skills at the age of about 4.5 years in the German context, we followed the recommendations in the handbooks of the respective measures for older children to estimate weak levels of phonological awareness. The procedure resulted in a group of *N* = 100 children. All children who performed above the 25th percentile were considered having normally to highly developed (in the following: normal) phonological awareness skills.

### Measures

#### Dependent variables

*Phonological awareness* was measured with a variety of subtests of phonological awareness in the broader and in the narrower sense. To identify children with weak phonological awareness skills at t_1_ (see above), scores in both of these domains were added up to a composite score.

The subtests used to assess phonological awareness were changed at t_3_, yet all subtests at t_1_/t_2_ and t_3_/t_4_, respectively, measure similar aspects of phonological awareness using the same or very similar item formats. The main reason for the change was to avoid possible ceiling effects in the subtests used at t_1_/t_2_, as children's phonological awareness skills develop rapidly in the age groups included in the study.

At t_1_ and t_2_ phonological awareness (broad) was measured with the following subtests from the Bielefelder Screening zur Früherkennung von Lese-Rechtschreibschwierigkeiten (Bielefeld screening for the early detection of *reading and spelling difficulties* = BISC; Jansen et al., 2002): Rhyme identification and Segmenting words into syllables. Each subtest contains 10 items, which means that a maximum of 20 points could be reached in this domain.

At t_3_ and t_4_ phonological awareness (broad) was measured with subtests from two different instruments: The subtest *Rhyming task* from the *Würzburger Vorschultest* (*Würzburg preschool test* = WVT; Endlich et al., 2016) as well as the subtests *Clapping syllables* and *Compounding syllables* from the phonological test *Rundgang durch Hörhausen* (= *Tour of the town Hörhausen*; Martschinke et al., 2011). Each of the three subtests has 8 items, which means that a maximum of 24 points could be reached in this domain.

At t_1_ and t_2_ phonological awareness (narrow) was measured with two subtests. First, the subtest *Sound-to-word* from the BISC (Jansen et al., 2002), which contains 10 items. Second, the subtest *Recognizing the initial sound* from the test *Aufgaben zur Erhebung der Phonologischen Bewusstheit im engeren Sinn* (*Exercises to assess phonological awareness in the narrower sense*; modified according to Marx and Weber, 2007), which has 8 items. In total, a maximum of 18 points could be reached in this domain.

At t_3_ and t_4_ phonological awareness (narrow) was measured with four subtests from the WVT (Endlich et al., 2016): *Recognizing the initial sound, Phoneme synthesis, Phoneme analysis*, and *Sound-to-word*. Each of these four subtests contains 8 items, which means a maximum of 32 points could be reached in this domain.

#### Control variables

Nonverbal cognitive abilities were measured at t_2_ using the subtest Nonverbal intelligence from the test Basisdiagnostik Umschriebener Entwicklungsstörungen im Vorschulalter—Version II (Basic diagnosis of specific developmental disorders in preschool—Version II = BUEVA-II; Esser and Wyschkon, 2012). The highest possible score in this subtest is 34 points.

*Working memory (visuo-spatial)* was assessed using an adapted version of the *Corsi-Block-Task* (Working Memory Test Battery for Children [WMTB-C]; Pickering and Gathercole, 2001). The highest possible value (*Corsi-Span*) equals 9.

*Musical competencies* were measured at t_1_ using the test *A Game for Understanding and Analyzing Your Child's Music Skills* (Audie; Gordon, 1989). Both subtests—one tapping the domain *Melody* and the other one tapping the domain *Rhythm*—were used. In total, a maximum of 20 points could be reached in this domain as each subtest includes 10 items.

Both the *receptive and productive components of the semantic lexicon* were measured at t_1_ using the corresponding subtests from the German version of the *Wechsler Preschool and Primary Scale of Intelligence* (WPSI-III; Petermann and Lipsius, 2009). The subtest *Passive (* = *receptive) lexicon* has 31 items and the subtest *Active (* = *productive) lexicon* has 26 items. In total a maximum of 57 points could be reached in this domain.

Grammatical competencies at t_1_ were measured with the subtest Composition of singular or plural forms as well as the subtest Repeating of sentences from the Heidelberger Sprachentwicklungstest (Heidelberg test on language development = HSET; Grimm and Schöler, 1991). In these two subtests, a maximum of 36 and 10 points could be reached, respectively.

The reliability of all aforementioned scales was satisfactory (all Cronbach's α ≥ 0.74).

#### Further background variables

The s*ocioeconomic status (SES)* was measured with the ISEI (= *International Socio-Economic Index of Occupational Status*) according to Ganzeboom et al. (1992). ISEI values can range from 16 (e.g., domestic helpers and cleaners) up to 90 (judges). For each family, the HISEI (= *Highest International Socio-Economic Index of Occupational Status*) was derived from the available ISEI coding scheme. If the ISEI value was only available for one parent, this value was used as the HISEI value for the family.

### Interventions

#### Training of musical competencies

In the participating preschools assigned to the condition with a consecutive combination of a musical and a phonological training (*n* = 10), a training program that aimed at promoting musical competencies was conducted by trained university students between t_1_ and t_2_ from January to May 2013. The training program is based on a German curriculum for early music education by Nykrin et al. (2007) called *Musik und Tanz für Kinder* (*Music and Dance for Children*). Exercises in the following eight domains were selected from this curriculum: meter, rhythm, pitch, basic music notation competencies, musical intervals and melody progression, dancing, collective singing, listening and playing of music, and joint drumming. Individual training sessions with a duration of about 20 min were conducted three times a week over a time period of 16 weeks. The training sessions were carried out in group settings with a maximum of five children per group. Every session started and ended with a ritual (e.g., a song). Additionally, every session included two active exercises, such as learning a new song, as well as two passive exercises, such as listening or dancing exercises. During the entire training period a stuffed animal called *Musikater* (*Music Cat*) served as a role model who led through the program by playfully announcing and explaining the exercises.

#### Training of phonological awareness

Training of phonological awareness took place in both the combination as well as the phonological training intervention group (*n* = 20 preschools) during the last year of preschool between t_3_ and t_4_ from January to June 2014. Here the combined version of the two editions of a well-established German training program called *Hören, lauschen, lernen* (*Hear, Listen, Learn*; HLL 1: Küspert and Schneider, 2008; HLL 2: Plume and Schneider, 2004) was implemented. As this training program is described in detail elsewhere (e.g., Schneider et al., 1997), the present article does not provide another comprehensive description.

The training program was conducted according to the guidelines of the manual, which recommended daily training sessions lasting about 10–15 min, to be held over a time period of 20 weeks. The training took place in group settings with an average of eight children and was conducted by the regular preschool teachers. A year earlier (in the beginning of 2013), these teachers were trained thoroughly by one of the authors of the training program. Additionally, the teachers practiced the implementation of the program by training another cohort of preschoolers during the previous year (2013). An additional booster session for the teachers took place right at the beginning of the training period (i.e., beginning of 2014).

## Results

To detect possible pretest differences among the training and control groups, an analysis of variance (ANOVA) was performed with the t_1_ measures of language (repeating sentences, singular/plural composition, receptive, and productive lexicon), working memory, nonverbal cognitive abilities, musical competencies, and the socioeconomic status. The results revealed no significant differences between the groups on any of these measures (all *p*s > 0.05). See Table 1 for means and standard deviations.

As the groups did not differ significantly on the control and background measures, none of them were included as covariates in the subsequent comparisons.

### Effects of musical training on phonological awareness (t_1_–t_2_)

To analyze the effects of the musical training on children's phonological awareness, a repeated measures analysis of variance (2 × 2 × 2) was conducted with two between-subject factors—training (yes vs. no), phonological awareness performance at t_1_ (weak vs. normal)—and the within-subject factor time (t_1_and t_2_). The analyses were performed separately for the two dependent variables phonological awareness (broad and narrow). For means and standard deviations see Tables 2, 3.

**Table 2:** **Scores on phonological awareness (broad) in percentage of correct answers (t_**1**_–t_**2**_)**.

	**Treatment group (music)**	**Performance group (t_1_)**	***M***	***SD***	***N***
Phonological awareness (broad) at t_1_	No	Normal	78.00	14.29	222
		Weak	51.49	13.21	74
		Total	71.38	18.12	296
	Yes	Normal	79.71	13.42	102
		Weak	46.16	17.01	26
		Total	72.90	19.59	128
	Total	Normal	78.54	14.02	324
		Weak	50.11	14.39	100
		Total	71.84	18.57	424
Phonological awareness (broad) at t_2_	No	Normal	83.92	15.23	222
		Weak	71.54	15.42	74
		Total	80.82	16.17	296
	Yes	Normal	77.99	16.87	102
		Weak	71.43	14.54	26
		Total	76.66	16.58	128
	Total	Normal	82.05	15.98	324
		Weak	71.51	15.13	100
		Total	79.57	16.39	424

**Table 3:** **Scores on phonological awareness (narrow) in percentage of correct answers (t_**1**_–t_**2**_)**.

	**Treatment group (music)**	**Performance group (t_1_)**	***M***	***SD***	***N***
Phonological awareness (narrow) at t_1_	No	Normal	51.09	18.05	222
		Weak	27.94	8.09	74
		Total	45.30	19.00	296
	Yes	Normal	52.35	16.27	102
		Weak	28.40	8.00	26
		Total	47.49	17.79	128
	Total	Normal	51.49	17.49	324
		Weak	28.06	8.03	100
		Total	45.96	18.65	424
Phonological awareness (narrow) at t_2_	No	Normal	59.06	20.55	222
		Weak	46.27	19.04	74
		Total	55.86	20.90	296
	Yes	Normal	61.43	20.95	102
		Weak	47.23	20.89	26
		Total	58.54	21.63	128
	Total	Normal	59.81	20.68	324
		Weak	46.52	19.43	100
		Total	56.67	21.14	424

The analysis of phonological awareness (broad) revealed that, overall, the groups improved from t_1_ to t_2_, resulting in a significant main effect of time, *F*_(1, 420)_ = 103.356, *p* < 0.001, $\eta_{p}^{2}$ = 0.19. There was no main effect of training, *F*_(1, 420)_ = 2.666, *p* > 0.05, nor was there an interaction between time and training, *F*_(1, 420)_ < 1, *p* > 0.05, indicating that the musical training had no effect on the development of phonological awareness (broad). However, there was an interaction between performance group (weak/normal) and time, *F*_(1, 420)_ = 71.46, *p* < 0.001, $\eta_{p}^{2}$ = 0.14. Apparently, the two performance groups differed in the development of their phonological awareness skills over time. The respective means show a more pronounced increase in the phonological awareness (broad) for the weak group compared to the normal group. Finally, and more interesting, there was a triple interaction between time, training and performance at t_1_,*F*_(1, 420)_ = 6.972, *p* < 0.01, $\eta_{p}^{2}$ = 0.01. This finding suggests that the two performance groups (weak/normal) developed differently in their phonological awareness skills over time, depending on whether they participated in the training or not. Contrasts between the weak and the normal group were performed comparing the training effects to break down this interaction.

For the children with normal phonological awareness at t_1_,a marginally significant effect of time was observed, *F*_(1, 322)_ = 3.574, *p* = 0.06, $\eta_{p}^{2}$ = 0.01. Moreover, there was a significant interaction of time and training, *F*_(1, 322)_ = 11.896, *p* < 0.01, $\eta_{p}^{2}$ = 0.03. The respective means show that the training group stagnated more or less in their performance in phonological awareness, whereas the control group improved slightly over time. A comparison of the gain scores (Δt_1_–t_2_) of the training group (*M* = 5.91; *SD* = 18.04) and the control group (*M* = −1.72; *SD* = 19.49) revealed that this difference was significant, *t*_(322)_ = 3.449, *p* < 0.001, *d* = 0.41.

Focusing on the children with weak phonological awareness, the analysis revealed a main effect of time, *F*_(1, 98)_ = 97.140, *p* < 0.001, $\eta_{p}^{2}$ = 0.49, indicating that both the training and the control group improved from t_1_ to t_2_. The interaction of time and training, however, turned out to be non-significant, *F*_(1, 98)_ = 1.289, *p* > 0.05, although the means showed that the training group started from an overall lower level at t_1_ and reached the same t_2_-level as the control group. The fact that this difference did not reach the level of significance is most likely due to the low statistical power caused by the small sample sizes of the respective weak groups (musical training: *N* = 26; control group: *N* = 74). In sum, there is no evidence that the training of musical skills improved phonological awareness (broad).

The same analyses were conducted to investigate the effect of musical training on phonological awareness (narrow). Again, the results show a main effect of time, *F*_(1, 420)_ = 106.523, *p* < 0.001, $\eta_{p}^{2}$ = 0.20, but no main effect of training, *F*_(1, 420)_ = 0.419, *p* > 0.05. Also, the interaction of time and training turned out to be non-significant, *F*_(1, 420)_ = 0.093, *p* > 0.05, indicating that, overall, the musical training had no effect on phonological awareness (narrow). There was a significant interaction of time and performance group (weak/normal) at t_1_, *F*_(1, 420)_ = 14.662, *p* < 0.001, $\eta_{p}^{2}$ = 0.03, but unlike the results of the former analysis, no three-way interaction of time, performance group, and training, *F*_(1, 420)_ = 0.013, *p* > 0.05. The means indicate that the weak performance group improved their phonological awareness skills (narrow) more than the normal performance group, on an overall lower level. However, this improvement seems to be independent from the training of musical competencies.

### Effects of phonological training and the combination of the two trainings on phonological awareness (t_3_–t_4_)

In a next step, we analyzed the development of phonological awareness from t_3_ to t_4_ for the three groups. As explained, the children who participated in the musical training subsequently received the phonological training (TG_music/phon_). Approximately half of the former control group also received the phonological training (TG_phon_) whereas the rest of the children formed the new control group (CG). Thus, a repeated measures analysis of variance (3 × 2 × 2) with two between subject factors—treatment condition (combined training, phonological training, no training), phonological awareness at t_1_ (weak vs. normal)—and the within subject factor time (t_3_ and t_4_) was performed. Again, the analyses were conducted separately for the dependent variables phonological awareness (broad) and phonological awareness (narrow). For means and standard deviations see Tables 4, 5.

**Table 4:** **Scores on phonological awareness (broad) in percentage of correct answers (t_**3**_–t_**4**_)**.

	**Treatment group**	**Performance group (t_1_)**	***M***	***SD***	***N***
Phonological awareness (broad) at t_3_	TG_music/phon_	Normal	56.22	16.14	102
		Weak	40.79	15.55	26
		Total	53.08	17.13	128
	TG_phon_	Normal	54.52	15.94	80
		Weak	47.88	21.57	29
		Total	52.76	17.76	109
	CG	Normal	57.14	14.19	142
		Weak	42.05	13.71	45
		Total	53.50	15.46	187
	Total	Normal	56.20	15.25	324
		Weak	43.41	16.87	100
		Total	53.19	16.55	424
Phonological awareness (broad) at t_4_	TG_music/phon_	Normal	67.73	16.62	102
		Weak	52.79	11.38	26
		Total	64.70	16.78	128
	TG_phon_	Normal	66.15	17.17	80
		Weak	58.55	16.92	29
		Total	64.13	17.35	109
	CG	Normal	67.85	14.75	142
		Weak	47.96	14.85	45
		Total	63.06	17.02	187
	total	Normal	67.39	15.94	324
		Weak	52.29	15.22	100
		Total	63.83	17.01	424

**Table 5:** **Scores on phonological awareness (narrow) in percentage of correct answers (t_**3**_–t_**4**_)**.

	**Treatment group**	**Performance group (t_1_)**	***M***	***SD***	***N***
Phonological awareness (narrow) at t_3_	TG_music/phon_	Normal	49.39	20.29	102
		Weak	33.89	18.16	26
		Total	46.24	20.77	128
	TG_phon_	Normal	52.25	19.77	80
		Weak	48.02	18.70	29
		Total	51.12	19.50	109
	CG	Normal	50.20	19.16	142
		Weak	37.41	14.56	45
		Total	47.12	18.93	187
	Total	Normal	50.45	19.64	324
		Weak	39.57	17.55	100
		Total	47.89	19.70	424
Phonological awareness (narrow) at t_4_	TG_music/phon_	Normal	64.25	18.91	102
		Weak	52.10	18.06	26
		Total	61.78	19.30	128
	TG_phon_	Normal	71.79	17.97	80
		Weak	65.27	17.62	29
		Total	70.06	18.03	109
	CG	Normal	60.85	17.98	142
		Weak	45.66	17.85	45
		Total	57.19	19.05	187
	Total	Normal	64.62	18.73	324
		Weak	53.02	19.51	100
		Total	61.89	19.53	424

**Figure 1:**
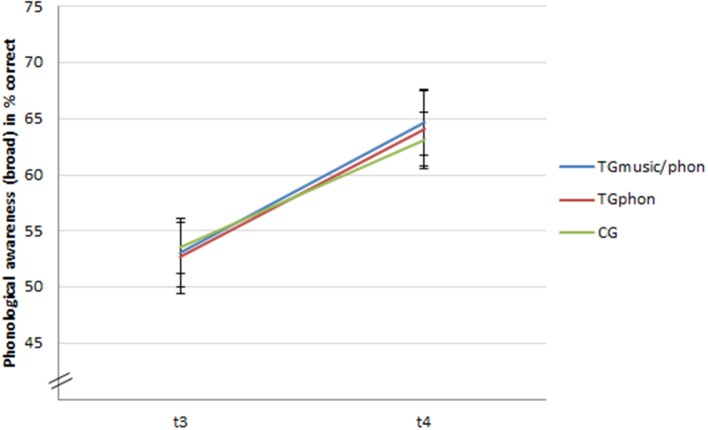
**Development ophonological awareness (broad) in percentage of correct answers (95% CI) from t_**3**_ to t_**4**_**.

The results revealed a main effect of time, *F*_(1, 418)_ = 129.139, *p* < 0.001, $\eta_{p}^{2}$ = 0.23, indicating that, overall, the groups improved their performance in phonological awareness (broad). All other effects turned out to be non-significant: main effect of training, *F*_(1, 418)_ = 1.348, *p* > 0.05; interaction of time × training, *F*_(2, 318)_ = 1.561, *p* > 0.05; interaction of time × performance group (weak vs. normal), *F*_(1, 418)_ = 0.922, *p* > 0.05; the three-way interaction of time × training × performance group (weak vs. normal), *F*_(2, 418)_ = 0.852, *p* > 0.05. Apparently, all groups progressed equally in their development of phonological awareness (broad) (see Figure 1). However, on a descriptive level (see Table 4), a clear trend is again observable: The weak groups gained more in the training conditions than in the control condition. Yet, probably due to the small sample sizes, this trend is not significant.

Finally, the effects of the training programs on phonological awareness (narrow) were compared with each other. There was a main effect of time, *F*_(1, 418)_ = 264.301, *p* < 0.001, $\eta_{p}^{2}$ = 0.38, as well as a main effect of training, *F*_(2, 418)_ = 11.497, *p* < 0.001, $\eta_{p}^{2}$ = 0.05. More interestingly, there was a significant interaction of time × training, *F*_(2, 418)_ = 10.376, *p* < 0.001, $\eta_{p}^{2}$ = 0.04. The interaction of time × performance group (weak vs. normal) as well as the three-way interaction of time × training × performance group (weak vs. normal) were both non-significant, *F*_(1, 418)_ = 0.059, *p* > 0.05, and *F*_(2, 418)_ = 1.016, *p* > 0.05.

**Figure 2:**
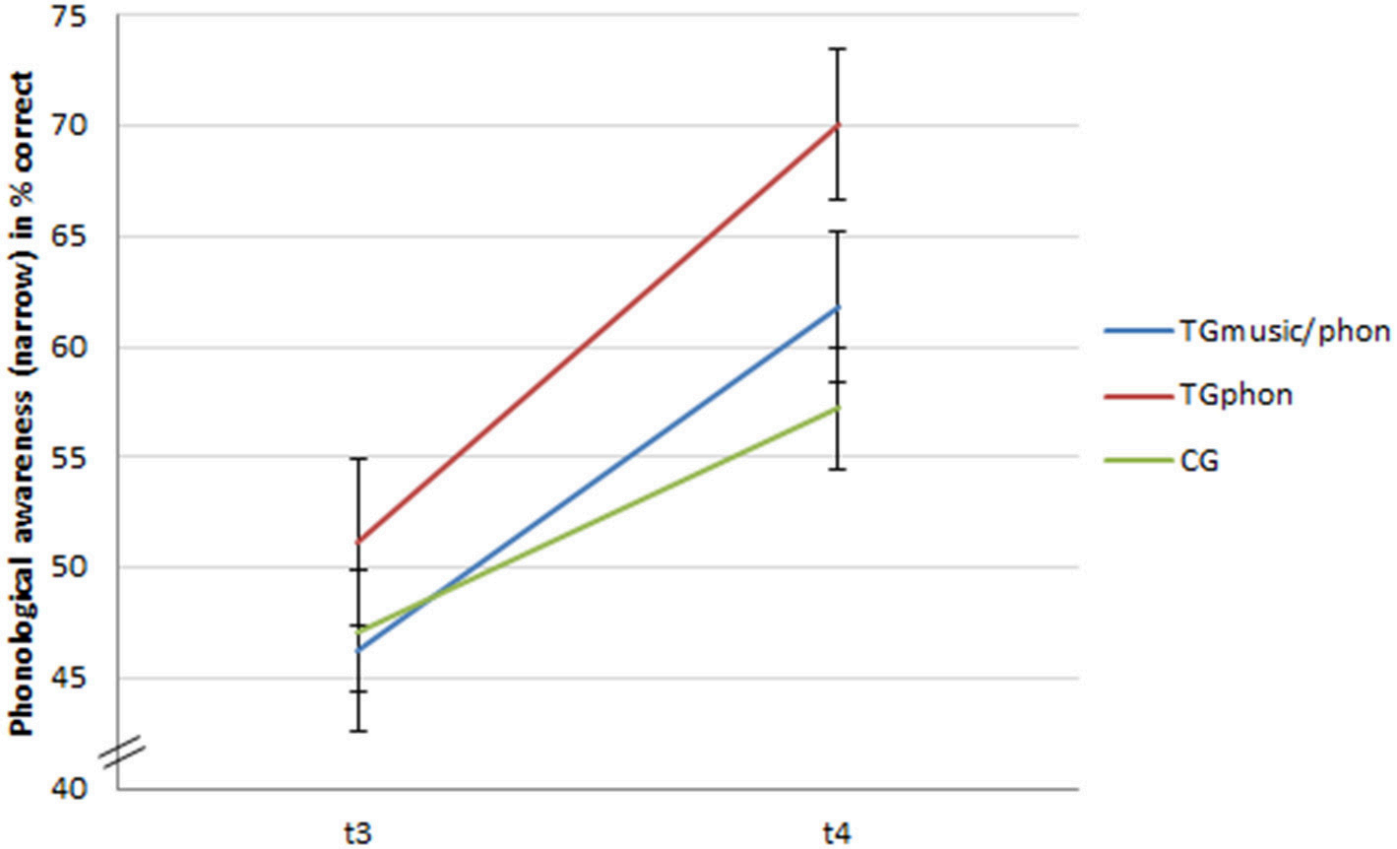
**Development of phonological awareness (narrow) in percentage of correct answers (95% CI) from t_**3**_ to t_**4**_**.

Planned contrasts were performed to break down the interaction of time and training between the two training groups and the control group. The comparison of the gain scores from t_3_ to t_4_revealed that the TG_phon_(*M* = 18.93, *SD* = 17.48) as well as the TG_music/phon_(*M* = 15.54, *SD* = 16.08) outperformed the CG (*M* = 10.06, *SD* = 13.81), *t*_(294)_ = 4.819, *p* < 0.001, *d* = 0.32, and *t*_(313)_ = 3.228, *p* < 0.001, *d* = 0.20. The two experimental groups, however, did not differ significantly *t*_(235)_ = 1.554, *p* > 0.05 (see Figure 2). This result indicates that the phonological training had an effect on phonological awareness (narrow) with a small to medium effect size.

### General analysis of training effects from t_1_ to t_4_

#### Analyses based on the whole sample

As there was a change in the instruments between the two training phases (between t_2_ and t_3_), it was not possible to model the development of phonological awareness across all four measurement points in one analysis. However, the extent to which the musical training had an effect over and above the effect of the phonological training cannot be tested with two subsequent pre-post analyses because each analysis controls for the respective pretest performances. Although detecting an incremental effect of the musical training is unlikely given the results from the first analysis, a multiple regression (OLS) was performed to determine if a delayed effect has occurred. Accordingly, we regressed phonological awareness at t_4_ using the two training groups as dummy-coded predictors (the control group represented the baseline) and controlling for the initial performance on phonological awareness in a next step. In addition, we entered musical competencies as well as grammatical competencies and lexicon in the model to investigate their relationship with phonological awareness and gain further insights regarding their relative importance for phonological awareness development. It can be assumed that these variables are very closely connected to phonological awareness and thus of central importance for the development of this domain. Again, separate analyses were conducted for the two domains of phonological awareness (broad and narrow) (see Tables 6, 7 for results).

**Table 6:** **Multiple linear regression (hierarchic) of phonological awareness (broad) in percentage of correct answers at t_**4**_ (***N*** = 424)**.

	**Model 1**		**Model 2**		**Model 3**		**Model 4**	
	***B (SE B)***	**β**	***B (SE B)***	**β**	***B (SE B)***	**β**	***B (SE B)***	**β**
Phonological training (1 = yes)	1.06 (2.05)	0.027	2.55 (1.75)	0.066	2.21 (1.73)	0.057	2.10 (1.68)	0.054
Musical and phonological training (1 = yes)	1.63 (1.95)	0.044	1.04 (1.66)	0.028	1.20 (1.63)	0.032	1.44 (1.59)	−0.039
Phonological awareness (broad) at t_1_			0.24 (0.04)	0.265^***^	0.21 (0.04)	0.236^***^	0.15 (0.04)	0.170^***^
Phonological awareness (narrow) at t_1_			0.34 (0.04)	0.383^***^	0.32 (0.04)	0.352^***^	0.24 (0.04)	0.267^***^
Musical competencies at t_1_					0.13 (0.03)	0.158^***^	0.09 (0.03)	0.110^**^
Grammatical competencies at t_1_							0.04 (0.05)	0.055
Lexicon at t_1_							0.22 (0.0)	0.216^***^
*R*^2^		0.002		0.286^***^		0.309^***^		0.353^***^

*** p < 0.001;

** *p < 0.01*.

**Table 7:** **Multiple linear regression (hierarchic) of phonological awareness (narrow) in percentage of correct answers at t_**4**_ (***N*** = 424)**.

	**Model 1**		**Model 2**		**Model 3**		**Model 4**	
	***B (SE B)***	**β**	***B (SE B)***	**β**	***B (SE B)***	**β**	***B (SE B)***	**β**
Phonological training (1 = yes)	12.86 (2.27)	0.288^***^	14.24 (2.12)	0.319^***^	13.80 (2.08)	0.309^***^	13.58 (2.04)	0.304^***^
Musical and phonological training (1 = yes)	4.59 (2.16)	0.108^*^	4.19 (2.00)	0.099^*^	4.39 (1.96)	0.103^*^	4.43 (1.92)	0.104^*^
Phonological awareness (narrow) at t_1_			0.25 (0.04)	0.245^***^	0.22 (0.04)	0.210^***^	0.12 (0.05)	0.123^*^
Phonological awareness (broad) at t_1_			0.22 (0.04)	0.214^***^	0.19 (0.04)	0.182^***^	0.12 (0.05)	0.115^*^
Musical competencies at t_1_					0.18 (0.04)	0.182^***^	0.14 (0.04)	0.141^**^
Grammatical competencies at t_1_							0.15 (0.06)	0.148^*^
Lexicon at t_1_							0.13 (0.07)	0.113^(*)^
*R*^2^		0.071^***^		0.212^***^		0.242^***^		0.280^***^

*** p < 0.001;

** p < 0.01;

* p > 0.05;

(*) *p < 0.10*.

The results of the regression analyses basically replicate the results of the pre-post comparisons in the repeated measures ANOVAs. Neither the combined training nor the phonological training alone had an effect on phonological awareness (broad) (Table 6, model 1). However, phonological awareness at t_1_ explains a considerable and significant proportion of variance in the criterion variable (28.4%; model 2). Musical competencies at t_1_ are also a significant predictor (model 3), which underlines the idea that phonological awareness and musical competencies are related during the early years of child development. Last and not unexpected, the language measures significantly explain further variance (model 4). This is, however, restricted to the lexicon; the predictive effect of grammatical competencies is not significant. Overall, the pattern reveals that pretest performance as well as language proficiency (lexicon) and, to a certain degree, musical competencies were the best predictors for later phonological awareness (broad) outcome. A formal training with musical and/or phonological contents as it was carried out in the present study was not successful at promoting phonological awareness (broad).

A different picture appears in the regression of phonological awareness (narrow) on the various predictors (Table 7). Both the combined training and the phonological training are significant predictors and explain 7% of variance. This is again in line with the pre-post analyses conducted before. However, the phonological training seems to be the better predictor with a β-weight more than twice the size as the β-weight of the combined training. This result holds true even after including the pretest performance in phonological awareness in model 2, which itself is a significant predictor explaining further 14.1% of variance. A comparable proportion of variance as in the regression of phonological awareness (broad) is explained by musical competencies (2.7%). This predictor is also statistically significant. Finally, in model 4 the language measures significantly explain further variance (3.8%). However, the pattern is reversed compared to the regression of phonological awareness (broad): now the grammatical competencies are a significant predictor whereas the predictive effect of lexicon is only marginally significant. In conclusion, the phonological training is the best predictor of phonological awareness (narrow) even after controlling for pretest performance, musical competencies as well as language measures. The effect of the combined training is also significant but smaller. In combination with the results of the pre-post analyses, the findings suggest that the effectiveness of the combined training is most likely due to the phonological training elements and not the musical training.

#### Analyses focusing on children with weak phonological awareness at the beginning of training

To further explore the third research question, the regression analyses were also performed for children with weak initial phonological awareness to detect whether this group benefits differentially from the training measures. The only difference to the regression analyses above is that phonological awareness at t_1_ is not included as a predictor in the model as the group was selected based on their pretest performance (below the 25th percentile). Once more, two separate regression analyses on phonological awareness (broad) and phonological awareness (narrow) were performed (see Tables 8, 9 for results).

**Table 8:** **Multiple linear regression (hierarchic) of phonological awareness (broad) in percentage of correct answers at t_**4**_ for the subsample of children with weak phonological awareness pretest scores (***N*** = 100)**.

	**Model 1**		**Model 2**		**Model 3**	
	***B (SE B)***	**β**	***B (SE B)***	**β**	***B (SE B)***	**β**
Phonological training (1 = yes)	10.59 (2.19)	0.317^**^	11.23 (3.20)	0.336^**^	10.60 (3.16)	0.318^**^
Musical and phonological training (1 = yes)	4.83 (3.62)	0.140	5.82 (3.31)	0.169^(*)^	6.08 (3.27)	0.176^(*)^
Musical competencies at t_1_			0.34 (0.07)	0.401^***^	0.29 (0.08)	0.342^***^
Grammatical competencies at t_1_					0.00 (1.10)	−0.005
Lexicon at t_1_					0.21 (0.11)	0.211^(*)^
*R*^2^		0.087^*^		0.223^***^		0.248 ^(*)^

*** p < 0.001;

** p < 0.01;

* p > 0.05;

(*) *p < 0.10*.

**Table 9:** **Multiple linear regression (hierarchic) of phonological awareness (narrow) in percentage of correct answers at t_**4**_ for the subsample of children with weak phonological awareness pretest scores (***N*** = 100)**.

	**Model 1**		**Model 2**		**Model 3**	
	***B (SE B)***	**β**	***B (SE B)***	**β**	***B (SE B)***	**β**
Phonological training (1 = yes)	10.94 (2.55)	0.252^***^	10.37 (2.48)	0.239^***^	11.10 (2.37)	0.256^***^
Musical and phonological training (1 = yes)	3.40 (2.37)	0.085	3.51 (2.30)	0.087	3.47 (2.21)	0.086
Musical competencies at t_1_			0.22 (0.05)	0.233^***^	0.13 (0.05)	0.141^**^
Grammatical competencies at t_1_					0.16 (0.7)	0.155^*^
Lexicon at t_1_					0.22 (0.85)	0.184^**^
*R*^2^		0.054^***^		0.108^***^		0.195^***^

*** p < 0.001;

** p < 0.01;

* *p > 0.05*.

In contrast to the results for the whole sample as well as the results of the respective pre-post analysis, it appears that children with weak phonological awareness skills benefit from the phonological training in their phonological awareness (broad). However, the musical training did not have any effect in this group. This is in line with the analyses for the whole sample. The marginally significant effect of the combined training assumingly stems from the phonological training elements. While the impact of the language variables also mirrors the pattern of results for the whole sample, it is noteworthy that musical competencies together with the phonological training are the strongest predictors in the regression of phonological awareness (broad) with medium effect sizes (model 3). This suggests that musical abilities are indeed closely related to phonological awareness, but the applied musical training program apparently failed to promote the relevant musical skills in the present sample.

The last regression analyses (see Table 9) reveals that the phonological training was also effective regarding phonological awareness (narrow). The combined training, however, had no effect on phonological awareness (narrow) indicating that both the phonological and the musical training together were not successful in this domain. The musical competencies as well as the language measures are all significant predictors (model 2 und 3) with small effect sizes. This is in line with the analyses for the whole sample.

## Discussion

The aim of the present study was to investigate the effects of a musical training in the penultimate year of preschool on the development of phonological awareness (broad and narrow) and whether its consecutive combination with a well-established phonological training in the last year of preschool would lead to incremental effects compared to a phonological training alone. This was conceptualized as a basis for a compensatory promotion of phonological awareness in children with weak initial phonological skills. Thus, a further goal of the study was to assess the training effectiveness for children with normally to highly developed and weak phonological skills separately to identify possible aptitude-treatment interactions. To this end, a longitudinal intervention study was conducted over a period of 2 years comparing the development of two different treatment groups with a control group.

With regard to the first research question on the effectiveness of the musical training, the results revealed that the applied musical training had virtually no effect on phonological skills in the penultimate year of preschool. This result holds true for both domains of phonological awareness (broad and narrow) as well as for both subsamples (children with normally and weak developed phonological skills). There was a tendency for the group with weak phonological skills to achieve higher gain scores in the musical training condition than in the control condition. Most likely due to the low test power, however, this difference did not reach the level of significance.

The analyses on the development of phonological awareness in the last year of preschool in the treatment groups TG_phon_ and TG_music/phon_ revealed the following results: For phonological awareness (broad) the data provided no evidence for a higher increase in skills in children of the training conditions, compared to children of the control condition. Descriptively, the means of the group with weak phonological skills seem to contradict this result as the training groups seem to outperform the control group. But again, the small sample sizes—implicating a low test power—did not suffice to reach the level of significance.

However, for the phonological awareness (narrow) the analyses showed a different picture. The significant interaction of time and training condition points to the effects of the training programs. The follow-up analyses confirmed that children of both training groups outperformed children of the control group. Contradictory to the hypotheses but in line with the former analyses regarding the musical training, the effects were more pronounced in the TG_phon_ than in the TG_music/phon_. Given that the musical training apparently was not effective, the small to medium-sized effects in this analysis might be explained by the impact of the phonological training elements in both treatment groups.

Taking into consideration that a change of test instruments was necessary between t_2_ and t_3_ in order to avoid ceiling effects, it was not possible to analyze the development of all groups across all four measurement points. Strictly speaking, the resulting two subsequent pre-post analyses are not adequate in detecting developmental differences between the groups from t_1_ to t_4_ since each analysis controls for the respective pretest scores. In order to consider this methodological weakness, the data were additionally examined using multiple regression analyses. In a first step phonological awareness at t_4_ (both domains—broad and narrow—separately) was regressed on various predictors using the whole sample. In a further step this procedure was repeated for the subsample of children with weak initial phonological awareness skills.

The results show that for phonological awareness (broad) the participation in the musical training or the combined training is not related to higher achievement scores at t_4_, compared to the control condition. This result holds true even after including phonological awareness at t_1_, musical competencies and language competencies as further predictors. All these variables, besides grammatical competencies, represent significant predictors—although with small effect sizes—explaining 35% of variance.

The analysis on phonological awareness (narrow) confirms the results of the pre-post analyses conducted before. Both training conditions (phonological and combined training) represent significant predictors. Together they explained 7% of variance. Their impact remained stable even after including the pretest scores of phonological awareness, musical competencies and language competencies in the final model. All of the predictors explained significant but small amounts of variance (altogether 28%). In this analysis it appears that the phonological training represents the strongest predictor with a medium-sized effect. On average, children in this group (TG_phon_) scored 13.5% higher on phonological awareness (narrow) than children in the control group (compared to 4.5% higher in the combined condition TG_music/phon_) whilst controlling for all other variables.

The same analyses were conducted with the subsample of children with weak initial phonological awareness skills (the only difference being that initial phonological awareness was not included as a predictor). For phonological awareness (broad) at t_4_ the results for this group suggest that the training conditions represented significant (phonological training) or marginal significant (combined training) predictors with medium and small effect sizes, respectively, explaining almost 9% of variance. This holds true after including all other predictors which in sum explained 25% of variance. Apart from the training effect in the weak performers, the initial musical competencies turned out to be the strongest predictor with a medium effect size in the final model whereas language competencies explained no or only marginal significant amounts of variance.

Last, the regression of phonological awareness (narrow) at t_4_ for the weak group revealed that the phonological training was again the strongest predictor in the controlled model whereas the influence of the combined training was non-significant. In this analysis musical competencies and language competencies contributed almost equally to the explanation of variance in phonological awareness with small but significant effects.

### Effects of the musical training

The present data provides no support for our first hypothesis that a musical training in the penultimate year of preschool leads to substantial gains in phonological awareness. This partly contradicts the results of previous research (e.g., Degé and Schwarzer, 2011; Moritz et al., 2013). At the same time the present study complements the meta-analysis by Gordon et al. (2015) revealing a very small (*d* = 0.2) overall effect size in presence of remarkable heterogeneity in the outcomes between the included studies. The main difference between the present study and the studies like the ones conducted by Degé and Schwarzer (2011) and Moritz et al. (2013) can be seen in the sample sizes, with that of the present study being considerably larger than those of the two other studies. Consequently, the results of our study seem more reliable in this regard. On the other hand, however, the participants in the current study were on average 1 year younger than those of most other experimental studies investigating the effects of early phonological awareness trainings. Theoretical considerations as well as results of other studies (Gathercole and Baddeley, 1993; Rothe et al., 2004) suggest that there might be a certain minimum age for the successful promotion of phonological awareness. This might also apply to the promotion of phonological skills by a musical training even though we hypothesized that the musical training should be language-dependent (and therefore age-related) to a lesser degree. Thus, the neuroplasticity necessary for refined brain responses to subtle speech cues may be decisively different between children of different age. This may be an explanation why some experimental studies with older children (e.g., aged 8–10) find effects of a musical training both on a behavioral and a electrophysiological level (e.g., François et al., 2013; Chobert et al., 2014) and even in children with developmental dyslexia (Flaugnacco et al., 2015).

A further explanation why no substantial effects were detectable refers to the fact that musical contents play a central role in the conventional course of preschools. Accordingly, the impact of a musical training has to outperform this “en-passant” training to become visible. The intensity of the musical training in the present study simply might have been too low.

The predictive value of musical competencies in the regression analyses independently from all other predictors indicates that musical competencies and phonological awareness are related constructs. This was especially noticeable in the group of children with weak initial phonological awareness scores. Additionally, the weak performing children tended to have higher gain scores in the musical training condition than the respective children in the control condition. These results indicate that this population might be expected to benefit most from a musical training under optimal circumstances.

### Effects of the phonological training and the combination condition

With respect to the second hypothesis regarding the effects of the phonological training, our assumptions were confirmed to some extent. In line with earlier research (Schneider et al., 1997; Schöppe et al., 2013), we found that the training is effective in promoting phonological awareness (narrow). The regression analyses provide further evidence that this also applies to the phonological awareness (broad) for the group with weak initial phonological awareness skills. The combination with a prior musical training, however, did not lead to incremental effects. Thus, this finding contradicts our second hypothesis. Quite surprisingly, the effect on phonological awareness was smaller in the TG_music/phon_ than in the TG_phon_. Given that the musical training showed no effects, we assume that the phonological training did in fact have an impact in this group which, however, was not as strong as in the TG_phon_. The reasons for this difference may be traced back to differences in the implementation quality which has been shown to be essential for training success (e.g., for phonological trainings Schneider, 2001). Generally, managing intervention quality is more challenging in studies with bigger samples (e.g., N > 100) compared to studies with smaller samples (e.g., N < 50) leading to overall increased variation in the outcomes and smaller effect sizes (Lipsey and Wilson, 1993). The available paper-pencil protocols which report attendance, deviance in the execution of the training, disturbances etc., do not provide indications of substantial quality differences but this possibility could only be ruled out by analyses of video material which is not available in this study.

### To which extent do children with weak initial phonological awareness skills benefit from training measures?

Regarding the question of how to best combine training measures in order to achieve compensatory effects in children with weak phonological prerequisites (research question 3), this study has no conclusive answer. The results provide only very limited evidence that a musical training at the age of about 4 years may be a promising measure to help children catch up with their peers. The pre-post analyses showed that the weak children in the musical training group did not outperform the weak children in the control group. After all, the well-established phonological training appears to be an effective way to generally help children develop phonological awareness skills, yet not to the extent that weak children reach the same performance level as children with initially normally developed phonological skills. This conclusion is substantiated by the fact that the three-way interactions which tested aptitude-treatment interaction effects were non-significant. The small sample sizes in the group of weak-performing children also reflects a possible problem of test power in the present study since the weak TG_phon_ descriptively outperformed even the normally developed CG in the domain phonological awareness (narrow). Regarding the testing of compensatory effects, the comparison of the weak-performing training groups to the normally developed control group would be the central comparison.

The divergent finding that children with weak prerequisites also benefit regarding phonological awareness (broad) reflects differences in the methods of analysis. In the repeated-measures ANOVA from t_3_ to t_4_ the focus was on the degree of change in phonological awareness only between these two points in time. In contrast, in the regression analysis the mean outcomes at t_4_ in different groups were compared given similar scores at t_1_. Since all groups were indeed comparable at t_1_ (this also holds true for the analyses on the whole sample) differences at t_4_ can plausibly be interpreted as training effects.

### Limitations of the present study

Some of the limitations of the present study have already been mentioned. From an experimental research point of view, a randomized design in which children are assigned to the experimental conditions individually would be preferable to the realized quasi-experimental design. However, in the context of research on young children this is hardly feasible. In a dynamic environment such as a preschool, the conduction of experimental research, especially over a period of 2 years with frequent interventions and extensive testing, is challenging by nature. Having a broad range of background information at hand to ensure comparability of the groups the present research can be seen as a combination of an experimental intervention study with a field approach. Under this perspective, we have the advantage of high ecological validity combined with minimum requirements of experimental research. Further, although a reasonable number of cases were included overall the groups of children with weak initial performances were too small to reliably detect significant effects. This problem could be addressed in future research by systematically oversampling this target group. A further point of criticism concerns the control group. A sound experimental design should use a treated control group to avoid differences between the groups in aspects such as motivation and amount of social interaction in order to validly differentiate experimental effects from mere observer effects.

The necessity to change the tests of phonological awareness between t_2_ and t_3_ in order to avoid possible ceiling effects restricts the interpretation of the results. There are good reasons to believe that the old and new instruments are actually measuring the same constructs in a very similar manner. However, small differences in the psychometric quality of the tests are likely. Therefore, a strict longitudinal data analysis was not possible. Since the evaluation of the data with repeated measurement and multiple regression analyses basically led to very similar results it can nevertheless be assumed that using the same instruments across all four measurement points would not have led to divergent patterns of results.

### Implications for future research and practice

There are some slight indications in the present study that musical skills and phonological awareness skills are indeed related. Therefore, the possibility of enhancing phonological skills by training musical competencies should not be ruled out. Thus, it is worth to address some aspects of the musical intervention to possibly increase training effectiveness in future research. First, given an optimal number of cases in a study, the intensity of a musical training could be manipulated. A pattern of a daily intervention over a period of 5 months—which is the framework of the phonological training program applied in the present study—could be seen as a benchmark for musical interventions (c.f. Gordon et al., 2015 for the role of trainings hours). The dynamic daily routines in preschools as well as the fluctuation of child presence due to sickness etc. can quickly lead to low individual training attendance rates. A constant input (e.g., daily) over an adequate period of time could compensate these risks. Furthermore, following the assumptions of a sensitive phase in the development of phonological awareness more research is needed to determine the optimal age for a musical intervention. It seems advisable to delay the beginning of training by about 1 year compared to the present study. The consecutive combination with a phonological training program is still possible in the last year of preschool and even a parallel implementation of both training programs is an option.

In addition to organizational considerations, the contents of the musical intervention may be altered. In the present study we chose a rather broad range of musical facets for intervention (comparable to Degé and Schwarzer, 2011). An emphasis on some aspects may be favorable. For instance, an emphasis on rhythm exercises is a promising approach considering the results of Moritz et al. (2013). Last, the consistent examination of implementation quality seems to be a further important step to successfully promote phonological awareness via musical contents. This especially holds true since the actual musical training is less standardized and evaluated as, for example, the well-established training of phonological awareness.

Until future research provides further evidence in favor or against a music-oriented approach, a well-established instrument of training phonological awareness is available for educational practice in German language (for English training programs c.f. Bus and van Ijzendoorn, 1999; Ehri et al., 2001). The presented data confirms that significant gains can be achieved especially in the domain of phonological awareness (narrow). It is suggested that these skills do not develop automatically but only through direct instruction, e.g., during early literacy classes. The effectiveness also applies to the domain of phonological awareness (broad) to a certain degree, namely when children start with weak phonological skills. Even if compensatory effects in children with weak initial skills are not guaranteed, the training of phonological skills contributes importantly to an early development of phonological awareness and in turn to a successful early literacy development. As musical contents play an important role in preschool anyway, practitioners can use the intuitive access and motivational nature of musical contents in addition to well-established training methods within a reflective approach to complementarily support the development of phonological awareness.

## Author contributions

SK: Substantial contributions to the acquisition, analysis, or interpretation of data; drafting the work; RG: Substantial contributions to the acquisition, analysis, or interpretation of data; partially drafting the work; revising the work critically for important intellectual content; KB: Substantial contributions to the acquisition, analysis, or interpretation of data; revising the work critically for important intellectual content; CT: Substantial contributions to the acquisition, analysis, or interpretation of data; revising the work critically for important intellectual content; CA: Substantial contributions to the conception or design of the work; revising the work critically for important intellectual content; WS: Substantial contributions to the conception or design of the work; revising the work critically for important intellectual content; PS: Substantial contributions to the conception or design of the work; revising the work critically for important intellectual content; All authors: final approval of the version to be published; agreement to be accountable for all aspects of the work.

## Funding

This research was funded by the Federal Ministry of Education and Research, award number 01GJ1205 A – C.

### Conflict of interest statement

The authors declare that the research was conducted in the absence of any commercial or financial relationships that could be construed as a potential conflict of interest.

## Footnotes

^1^In the German context ”Kindergarten“ corresponds to preschool/child care centers in the U.S. education system. Starting Kindergarten at around three years of age, children normally leave Kindergarten by the age of six years to enter elementary school. We use the term preschool within the meaning of the German term Kindergarten throughout this article.
